# On Nelaton’s Method of Resuscitation from Chloroform Narcosis

**Published:** 1874-10

**Authors:** J. Marion Sims

**Affiliations:** Surgeon to the Woman’s Hospital of the State of New York, etc.


### ON NELATON'S METHOD OF RESUSCITATION FROM CHLOROFORM NARCOSIS.

By J. MARION SIMS, M.D.,
*Surgeon to the Woman's Hospital of the State of New York, etc.*

Read at the Forty-second Annual Meeting of the British Medical Association, held in Norwich, August, 1874.

Dr. Charles James Campbell, the distinguished accoucheur of Paris, has recently written two papers on anæsthesia in obstetrics, in which he ably sustains the views, long taught by Nelaton, that death from chloroform is due to syncope or cerebral anæmia; and, amongst other strong arguments to prove his position, he gave a graphic description of a case of chloroform narcosis, which occurred in my practice in Paris, where M. Nelaton, by his method, unquestionably saved the life of the patient. She was young, beautiful and accomplished, and belonged to one of the oldest and best families of France. Married at twenty, she gave birth to her first child a year afterwards. The head was enormous (hydrocephalic), impacted in the pelvis nearly twenty-four hours, and the delivery of a dead child was ultimately accomplished with instruments. Dr. Bouchacour, of Lyons, was called in consultation, and applied the forceps. In a week afterwards the urine began to dribble away, and in a fortnight an immense slough was thrown off. The case, surgically considered, was one of the most interesting I ever saw, and the operation was one of the most difficult I ever performed on any one in her station in life. The base of the bladder was destroyed, and the fundus fell through the fistulous opening; it was therefore inverted, and protruded between the labia majora as a herniary mass of the size of an apricot, its external covering being the internal or lining membrane of the bladder, which was of a deep vermillion red color. The vaginal portion of the cervix uteri and the posterior cul-de-sac were destroyed; and, by the reparative process, the cervix and the posterior wall of the vagina were blended into one common cicatrical mass, which was firm, inelastic, and immovable. The case appeared desperate, and M. Nelaton had pronounced it incurable. A preparatory operation was necessary, viz.: to open the cervix uteri, by dissecting it from the posterior wall of the vagina, and thus to reconstitute the canal of the vagina up to the canal of the cervix, and, by a subsequent operation, to draw forward the flap thus formed, secure it to the neck of the bladder anteriorly, and thereby close the fistula. The first, or preparatory operation, was performed at the country house of the family, near Dijon, on November 3d, 1861, Dr. Dugast, of Dijon, assisting, and giving chloroform. The second, or operation for the radical cure, was performed on the 19th of the month, at St. Germain, about an hour's distance from Paris by rail. M. Nelaton, Dr. Campbell, Dr. Beylard, Dr. Johnston, and Mr. (now Dr.) Alan Herbert, were present. I seldom give an anæsthetic in private practice for operation on the walls of the vagina, as the pain is generally not sufficient to call for it; but in this case, as the slightest touch was unbearable, an anæsthetic was indispensable. Dr. Campbell was selected by the family, as well as by M. Nelaton and myself, to administer the chloroform, especially as he was in the daily habit of giving it in his large obstetric practice, and we all had entire confidence in his caution, skill and judgment. The patient was soon anæsthetised. The operation was begun at 10 A.M., and I thought it would require about an hour to finish it.

Many years ago I imbibed the convictions of my countrymen against chloroform in general surgery, and have always used ether in preference, never feeling the least dread of danger from it under any circumstances. It is otherwise with chloroform, and in this particular case I felt the greatest anxiety, frequently stopping during the operation to ask Dr. Campbell if all was going on well with the patient. At the end of forty minutes the sutures (twelve or thirteen) were all placed and ready to be secured, and I was secretly congratulating myself that the operation would be finished in a few minutes more, when all at once I discovered an unusual bluish livid appearance of the vagina, as if the blood were stagnant, and I called Dr. Johnston's attention to it. As this lividity seemed to increase, I felt rather uneasy about it, and I asked Dr. Campbell if all was right with the pulse. He replied, "All right—go on." Scarcely were these words uttered, when he suddenly cried out, "Stop! stop! no pulse, no breathing!" and, looking to M. Nelaton, he said, "Tete en bas, n'est-ce pas?" Nelaton replied, "Certainly—there is nothing else to do." Immediately the body was inverted, the head hanging down, while the heels were raised high in the air by Dr. Johnston, the legs resting, one on each of his shoulders. Dr. Campbell supported the thorax. Mr. Herbert was sent to an adjoining room for a spoon, with the handle of which the jaws were held open, and I handed M. Nelaton a tenaculum, which he hooked into the tongue, and gave in charge to Mr. Herbert, while to Dr. Beylard was assigned the duty of making efforts at artificial respiration, by pressure alternately on the thorax and abdomen. M. Nelaton ordered and overlooked every movement, while I stood aloof and watched the proceedings with, of course, the most intense anxiety. They held the patient in this inverted position for a long time before there was any manifestation of returning life. Dr. Campbell, in his report, says it was fifteen minutes, and that it seemed an age. My notes of the case, written a few hours afterwards, make it twenty minutes. Be this as it may, the time was so long that I thought it useless to make any further efforts, and I said, "Gentlemen, she is certainly dead, and you might as well let her alone." But the great and good Nelaton never lost hope, and, by his quiet, cool, brave manner, he seemed to infuse his spirit into his aids. At last there was a feeble respiration, and after a long time another, and by-and-bye another; and then the breathing became pretty regular, and Dr. Campbell said, "The pulse returns, thank God; she will soon be all right again." Dr. Beylard, who always sees the cheerful side of everything in life, was disposed to laugh at the fear I manifested for the safety of our patient. I must confess that never before or since have I felt such a grave responsibility. When the pulse and respiration were well re-established, M. Nelaton ordered the patient to be laid on the table. This was done gently. But what was our horror when, at the moment the body was placed horizontally, the pulse and breathing instantly ceased. Quick as thought, the body was again inverted, the head downwards, and the feet over Dr. Johnston's shoulders, and the same manœuvres as before were put in execution. Dr. Campbell thinks it did not take such a long time to re-establish the action of the lungs and heart as in the first instance. It may have lacked a few seconds of the time, but it seemed to me to be quite as long; for the same tedious, painful, protracted and anxious efforts were made as before, and she seemed, if possible, more dead than before; but, thanks to the brave men who had her in charge, feeble signs of returning life eventually made their appearance. Respiration was at first irregular, and at long intervals; soon it became more regular, and the pulse could then be counted, but it was very feeble, and would intermit. I began again to be hopeful, and even dared to think that at last there was an end of this dreadful suspense, when they laid her horizontally on the table again, saying, "She is all right this time." To witness two such painful scenes of danger to a young and valuable life, and to experience such agony of anxiety, produced a tension of heart and mind and soul that cannot be imagined. What, then, must have been our dismay, our feeling of despair, when, incredible as it may seem, the moment the body was laid in the horizontal position again, the respiration ceased a third time, the pulse was gone, and she looked the very picture of death? Then I gave up all as lost; for I thought that the blood was so poisoned, so charged with chloroform, that it was no longer able to sustain life. But Nelaton, and Campbell, and Johnston, and Beylard, and Herbert, by a consentaneous effort, quickly inverted the body a third time, thus throwing all the blood possible to the brain, and again they began their efforts at artificial respiration. It seemed to me that she would never breathe again; but at last there was a spasmodic gasp, and, after a long while, there was another effort at respiration; and, after another long interval, there was a third; they were "far between;" then we watched, and waited, and wondered if there would ever be a fourth; at length it came, and more profoundly, and there was a long yawn, and the respiration becamc tolerably regular. Soon Dr. Beylard says, "I feel the pulse again, but it is very weak." Nelaton, after some moments, ejaculates, "The color of the tongue and lips is more natural." Campbell says, "The vomiting is favorable; see, she moves her hands; she is pushing against me." But I was by no means sure that these movements were not merely signs of the last death-struggle, and so I expressed myself. Presently Dr. Johnston said, "See here, doctor; see how she kicks; she is coming round again;" and very soon they all said, "She is safe at last." I replied, "For heaven's sake, keep her safe; I beg you not to put her on the table again till she is conscious." This was the first and only suggestion I made during all these anxious moments, and it was acted upon; for she was held in the vertical position till she, in a manner, recovered semi-consciousness, opened her eyes, looked wildly around, and asked what was the matter. She was then, and not till then, laid on the table, and all present felt quite as solemn and as thankful as I did; and we all in turn grasped Nelaton's hand, and thanked him for having saved the life of this lovely woman.

In a few minutes more the operation was finished, but of course, without chloroform. The sutures were quickly assorted and separately twisted, and the patient put to bed; and, on the eighth day thereafter, I had the happiness to remove the sutures in the presence of M. Nelaton, and to show him the success of the operation.

I have detailed the circumstances of this interesting case at great length, because I believe it goes as far to establish a principle of treatment as any one case ever did, or possibly can.

If the recovery had been complete and perfect with the first effort at reversing the body, there might have been a doubt whether the vertical position was really the cause of resuscitation; but, when the horizontal position was again and again followed by a cessation of all evidence of life, and when life was again and again re-established by a position that favored only the gravitation of the blood (poisoned as it was) to the brain, the inference is very clear that death in such cases is due to syncope, or cerebral anæmia. Exhaust the brain of blood in any way, and death follows. Fill it speedily with blood again, and life returns.

I have another case to relate, which goes far to establish the principle of treatment in chloroform narcosis, so forcibly illustrated by the case at St. Germain.

In January, 1873, I amputated the cervix uteri at the Woman's Hospital, drew the vaginal tissue over the stump, and secured it by silver sutures. The junior house-surgeon gave the anæsthetic. When the operation was nearly finished, he cried out, "The patient has stopped breathing," and immediately added, "She has no pulse." As before stated, I always use ether as an anæsthetic, and could not realize the fact that my patient was in any danger whatever, until I was told that they were giving her a mixture of chloroform and ether (one part to four,) which some of the surgeons had been using a few days previously. On examining the patient, I found her, as it were dead; there was not the slightest muscular rigidity; the arms and head fell by their own gravity in any way they were directed; the neck was as limber as if it were a mere band of soft linen stretching from the head to the trunk; there was not the least sign of breathing or of the pulse; she was, to all intents and purposes, dead; and I believe she would certainly have remained so if she had been left alone; and I doubt very much whether she could possibly have been resuscitated by any other method than that of Nelaton.

I quickly inverted the body, and had it held thus; and then I shook the thorax, agitating the head laterally, so as to add an impetus to the movement of the blood, which, with the body in this vertical position, would naturally gravitate toward the brain; the jaws were held asunder, and the tongue hooked with a tenaculum, and pulled forward; in a few minutes the breathing was re-established, and then the pulse returned; and soon the patient was placed again on the table in a lateral semi-prone position, in which all my operations on the uterus are performed; and the operation was finished, but without any more of the anæsthetic.

These two cases comprise my personal experience with Nelaton's method in chloroform narcosis.

The *New Orleans Medical and Surgical Journal*, for November, 1873, says: "In the course of an extended experience in the administration of chloroform, it has happened three times to Dr. M. Schuppert, that, to all appearances, the narcotized subject died—that is, respiration ceased, the heart stopped beating, and muscular contractility became extinct. The method he adopted for resuscitating these patients consisted in reversing the body, either by hanging them up by the feet, or laying them over a bed or table, so that the greater part of the body, with the head, hung down. In that position, artificial respiration was also tried. In one case, five minutes elapsed before there was a natural inhalation. All of them recovered. Dr. Schuppert believes that in cases of death from chloroform, the primary cause of the cessation of the respiration and circulation rests in anæmia of the brain, and not in impregnation of the blood with carbonic acid."

Another American authority, Dr. E. L. Holmes (*Chicago Medical Journal*, September, 1868,) says that whenever there is any failure of the heart's action, as is nearly always the case, the body should be laid at an angle of 40°, with the head downward so as to favor the passage of arterialized blood to the brain.

I take it for granted that Dr. Schuppert and Dr. Holmes must have obtained their knowledge of this method of resuscitation either directly or indirectly from the teachings of Nelaton; for he had for years been in the habit of explaining his method in his lectures and at his *cliniques*, and Dr. Johnston published an account of it in the American papers in 1861. Ten years ago there was a story prevalent in Paris that M. Nelaton had derived the hint of reversing the body in chloroform-poisoning from a discovery accidentally made by his little son, then some seven or eight years old; that, without thought or reason, he had taken up a dead mouse by the tail, and was twirling it around, when, to his surprise, it began to manifest signs of life, and recovered entirely, while the mice left lying were dead; and that the great surgeon was thus taught a great lesson, if not by babes and sucklings, at least by a little boy. This is a very pretty story, as it is, and it seems a pity to spoil it. A few days ago, when in Paris, I called to see young Nelaton (who is now a student of medicine, and will graduate next year), and I asked him for the facts of the mouse story. He said that when they lived on the Quai Voltaire, the house was infested with mice; that great numbers were caught in traps almost daily; that he was in the habit of killing them with chloroform by covering the trap with a napkin and pouring the chloroform on it; and that his only idea was that of an easy death for the mice. One day, when he had given a happy dispatch to some mice, his father accidentally came into the room, and, seeing the dead mice, he told his son if he would take up one by the tail, and hold it with the head downwards, that it would revive, while the others would not. He did this, and found it was true. And he told me that he had, when a boy, performed the same experiment on mice some forty or fifty times, or more, and always with the same unvarying result. He says that he has often heard his father speak, not only of the case that occurred at St. Germain, but of other cases that he had saved in the same way before the time of the mouse story, which dates back to 1857 or 1858.

As the facts now laid before you fully explain themselves, it is unnecessary for me to indulge in any lengthened remarks on the subject. In my own country, the accoucheurs often use chloroform, and the surgeons mostly use ether. I believe there has not as yet been a single death from chloroform given during labor, while deaths from it in general surgery occur constantly, and for unimportant operations. There must be a reason for this. I believe it can be explained only on the theory that death from chloroform is, as a rule, due to syncope or cerebral anæmia. Now, we know that in active labor there can be no cerebral anæmia, for every pain throws the blood violently to the head, producing fullness and congestion of the blood-vessels, thereby counteracting the tendency of the chloroform to produce a contrary condition. It may be said that the recumbent position has some influence in determining the safety of chloroform in labor; and so it has, but it gives no immunity under other circumstances. Chloroform, given intermittingly, as in labor, is thought to be less dangerous; but patients in labor are often kept for hours under its influence with safety, and occasionally it is necessary to produce complete and profound narcosis in some obstetrical operations; and yet, I believe, I can safely reiterate, what I have already said, that no woman has as yet died in labor from the effects of this anæsthetic. In puerperal convulsions, where the brain is believed to be overcharged with blood—and that, too, when the blood is known to be poisoned with urea—we formerly bled the patient, and we do so now sometimes, but our chief remedy is chloroform, which acts by arresting spasmodic movements, and by producing that very state of cerebral anæmia so necessary to a successful result. Whether puerperal convulsions are less frequent in labors under chloroform than in those without it, I do not know.

I believe that obstetrics may take a lesson from Nelaton's method of resuscitation, by adopting it in cases of threatened death from *post partum* hæmorrhage. Let us not be satisfied with simply placing the head low, but let us, in addition to the means usually adopted, invert the body, and throw what little blood there is left in it wholly to the brain. I have never seen a death from uterine hæmorrhage, but, from recollections of the few alarming cases I have witnessed, I now feel sure that recovery might have been hastened if I had known of and adopted Nelaton's method of inversion.

Whether death from chloroform is due to cerebral anæmia or not, it is at least safe to adopt Nelaton's method in all cases of supposed or threatened danger; but I think the safest plan is to relinquish the use of chloroform altogether, except in obstetrics. The frequent cases of death from the use of chloroform in surgical operations that have occurred amongst us, even of late, should warn us to give up this dangerous agent, if we can find another that is as efficient and, at the same time, free from danger. Ether fulfils the indications to a remarkable degree; but, while it is safe, it is unfortunately unpleasant to the physician and bystanders, as well as to the patient. He who will give us an anæsthetic as pleasant to take as chloroform, and as safe as ether, will confer the greatest boon upon science and humanity.

